# Digitized Seedbed Soil Quality Assessment from Worn and Edge Hardened Cultivator Sweeps

**DOI:** 10.3390/s24216951

**Published:** 2024-10-29

**Authors:** Jong-Myung Noh, Lijie Liu, Mehari Z. Tekeste, Qing Li, Jerry Hatfield, David Eisenmann

**Affiliations:** 1 Department of Agricultural and Biosystems Engineering, Iowa State University, Ames, IA 50011, USA; jnoh@iastate.edu; 2 Department of Industrial and Manufacturing Systems Engineering, Iowa State University, Ames, IA 50011, USA; lijiel@iastate.edu (L.L.); qlijane@iastate.edu (Q.L.); 3 Department of Agronomy, Iowa State University, Ames, IA 50011, USA; jerryhatfield67@gmail.com; 4 Department of Materials Science and Engineering, Iowa State University, Ames, IA 50011, USA; djeisen@iastate.edu

**Keywords:** digital tillage, LiDAR, precision tillage, seedbed quality

## Abstract

Tillage tools for seedbed soil management are often subjected to low stress abrasion wear, which could negatively affect seedbed quality and crop productivity. Limited studies exist that quantify the effects of worn tillage tools on seedbed quality and crop yield. This research investigated the influence of tillage tool wear on seedbed preparation by evaluating the effect of cultivator sweep wear on soil tilth utilizing a light detection and ranging (LiDAR) sensor. The framework consists of a seedbed tillage field experiment using a Completely Randomized Design (CRD) experiment in six replicates of two-tillage treatments (new and worn cultivator sweeps). After seedbed tillage, loosely tilled soil aggregates were removed to expose the seedbed soil profile, and then seedbed roughness statistical measures were estimated from LiDAR-scanned seedbed soil surface. Three statistical analyses (Analysis of Variance (ANOVA), Kolmogorov–Smirnov (KS), and Earth Mover’s Distance (EMD)) were compared to quantitatively evaluate the soil roughness estimated from the LiDAR seedbed surface data. Seedbed prepared by new and worn cultivator sweeps showed significant differences (*p* < 0.05) in soil roughness variables of standard deviation, coefficient of variation, and kurtosis. Data analysis from the ANOVA and KS methods revealed that LiDAR-extracted soil roughness patterns were statistically influenced by tillage treatment. EMD analysis detected noticeable disparities between the tillage treatments and new versus worn cultivator sweeps. This study concludes that tillage tool wear substantively affects seedbed quality, as evidenced by LiDAR soil profile estimated attributes of soil roughness and three statistical methods (ANOVA, KS, and EMD). Our study supports the adoption of LiDAR technology for seedbed management, highlighting its applicability to evaluate seedbed quality that accounts for the wear life cycle of cultivator sweeps.

## 1. Introduction

Precision tillage is essential in preparing soil for planting, crop emergence, crop growth, and maximizing the yield during the agricultural crop production cycle. Tillage practices impact crop growth factors, including both the crop emergence and their uniformity of seed depth [1]. The tillage practices often involve primary and secondary tillage implements that are designed for utilizing tillage ground engaging tool type and equipment settings to achieve the desired soil conditions for maximizing yield while minimizing tillage fuel consumption. In traditional farming practices, secondary tillage for seedbed preparation and the soil-engaging components of planters affect seedbed quality, which in turn affects seed placement accuracy, crop emergence, crop growth, crop yield, and the environment, such as runoff and soil erosion. A well-prepared seedbed can be described as a uniform surface and soil conditions that promote the desired soil-to-seed contact and enhance the expected outcomes of planting operations in terms of uniform seed depth, seed spacing, and uniform plant emergence [2].

Assessing the seedbed quality is often challenging due to the influence of factors such as tillage tool design, operation speed, depth, and field soil conditions (soil type, moisture content, and residue cover) [3]. Soil roughness is one of the soil tillage quality indicators that defines the soil tilled surface conditions after tillage tools pass. Traditional methods such as pinboard [4] and a chain method [5] exist to evaluate the soil roughness by physically placing them on the measurement area. The pinboard and chain methods for measuring soil roughness are prone to manual measurement errors and have limitations for covering the entire measurement area. Digital and automated assessments of soil roughness provided an improved depiction of the spatial differences of soil surfaces in both natural environments and agricultural systems [6]. Over the last few decades, many studies have deployed digitized soil surface evaluation methods that have relevance for soil tillage management. Ball et al. (2017) conducted research on visual soil evaluation techniques, which could prove useful in determining changes in soil roughness resulting from soil management practices and compaction [7]. Others have also developed sensor-based methods for assessment of soil roughness using various sensors, such as terrestrial laser scanners [8], cameras [9], and light detection and ranging (LiDAR) sensors [10]. Fanigliulo et al. (2020) conducted a study on soil surface detection using 3D imaging techniques with a light drone to assess soil roughness [11] for comparing various tillage methods. According to Foldger et al. (2019), the LiDAR-based method exhibited high resolution for spatial data acquisition and consistency in the results due to its noncontact and automated process as compared to traditional methods [10,12]. The previous studies that deployed digital data acquisition and visualization techniques have not considered the relationship between the soil tilth quality that accounts for tillage wear-induced alteration of tool geometries because the tilled soil conditions are affected by the soil-to-tool interaction, soil types, and soil conditions (e.g., soil wetness) [13]. With the advancement of software and hardware integration on soil tillage equipment and management systems, equipment manufacturers offer to growers ISOBUS with sensors embedded to the ground engaging tools of the tillage equipment. For example, Case IH, an equipment manufacturer, has developed a new technology and integrated software, Advanced Farming Systems (AFS) Soil Command™ (Racine, WI, USA) with ISOBUS connectivity to operator display and connected to a linear spring sensor on the ground engaging shank. Using the AFS Soil Command™, farmers can adjust a field cultivator sweep’s vertical position in real time during tillage operation to achieve the optimum seedbed condition. Current tool position adjustment from the operator display and control feedback system of the AFS Soil Command™ requires further improvement as the current system does not account for how worn tillage tools can affect soil seedbed quality. Improvement of the control feedback is needed for better assessments of agricultural productivity. Furthermore, investigating the outcome of tillage soil management due to the wear of tillage tools is of paramount importance. This is because the effect of tool wear is significant not only on tillage quality but also on the replacement cost of tillage tools, energy consumption during tillage operation, and ultimately the overall cost of the production, which is related to the farmer’s profit [14,15,16]. Limited studies are available to investigate the tillage tool wear effects on seedbed quality. The overall goal of this study is to provide a better understanding of soil management and tillage tools that experience abrasive wear and valuable insights to crop growers to facilitate effective decision-making regarding tillage wear characteristics and edge-hardening technologies on tillage tools as seedbed soil management.

The objectives of this study are to (1) evaluate seedbed quality using 2D LiDAR technology by assessing soil roughness attributes and (2) investigate cultivator sweep wear effects on seedbed quality by using new and worn cultivator sweeps on seedbed formation.

## 2. Materials and Methods

### 2.1. Experimental Site Description

The seedbed tillage experiment was conducted at a research site (approximately 8 ha), located at 41°58′ N and 93°40′ W, close to the Agricultural Engineering and Agronomy Research (AEA) farm of Iowa State University (ISU) in Boone, Iowa. According to the USDA soil survey (https://websoilsurvey.sc.egov.usda.gov/app/WebSoilSurvey.aspx accessed on 30 June 2024), Clarion loam (*fine-loamy*,* mixed*,* super-active*,* mesic Typic Hapludolls*), Canisteo clay loam (*fine loamy*,* mixed*,* superactive*,* calcareous mesic Type endoqualls*), Nicollet (*fine-loamy*,* mixed*,* superactive*,* mesic Aquic Hapludolls*), Webster (*fine-loamy*,* mixed*,* superactive*,* mesic Typic Endoaquolls*), Okoboji (*fine*,* smectitic*,* mesic Cumulic Vertic Endoaquolls*), and Harps (*fine-loamy*,* mixed*,* superactive*,* mesic Typic Calciaquolls*) with percent area coverage of 32.4%, 26.1%, 23.0%, 6.7%, 5.4%, and 6.4%, respectively, are the dominant soil series at the field plot.

### 2.2. Tillage Experimental Design and Equipment

**Figure 1:**
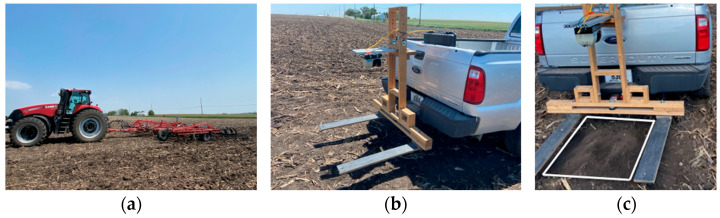
Case IH Magnum 380 row crop tractor equipped with (**a**) Case IH Tiger-Mate 255 Field Cultivator; (**b**) a pickup truck mounted with SICK LiDAR sensor (LMS 511-10100 PRO SR) (Minneapolis, MN, USA) and on the wood reference frame for data collection; and (**c**) seedbed surface designated for SICK LiDAR scanning area (0.77 m wide and 0.83 m long).

**Figure 2:**
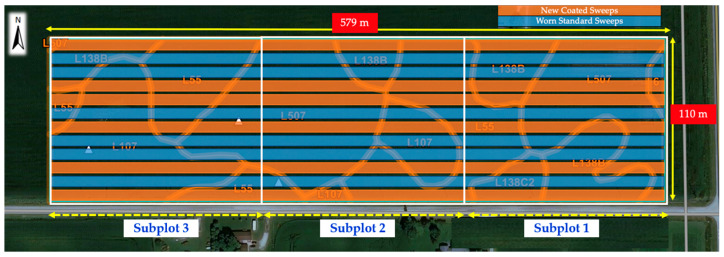
A CRD experimental design of the tillage experiment showing the twelve tillage passes with new cultivator sweeps (blue) and worn cultivator sweeps (orange). It was separated into three subplots (three white boxes) to evaluate the dominant soil type effects on tillage quality. The base soil map layer of the site was from the USDA soil survey (https://websoilsurvey.sc.egov.usda.gov/app/WebSoilSurvey.aspx accessed on 30 June 2024).

We investigated the effects of two tillage tools on seedbed quality by using new and worn cultivator sweeps on field cultivator equipment, Case IH Tiger-Mate 255 Field Cultivator (Racine, WI, USA) pulled by Case IH Magnum 380 row crop tractor (Racine, WI, USA) (Figure 1). A tillage operation was conducted using a Completely Randomized Design (CRD) in six replicates of the new and worn cultivator sweeps. Each replicate consisted of field cultivation performed by the Case IH Tiger-Mate 255 Field Cultivator, one with a set of new coated cultivator sweeps and the other with a set of worn cultivator sweeps. Each of the tillage treatment field plots was divided into three subplots, each measuring 189 m by 110 m for data sampling. The experimental field (579 m long by 100 m wide) layout for the three subplots and the two tillage treatments in six replicates is shown in Figure 2.

### 2.3. Geometric Dimensions of New and Worn Cultivator Sweeps

**Figure 3:**
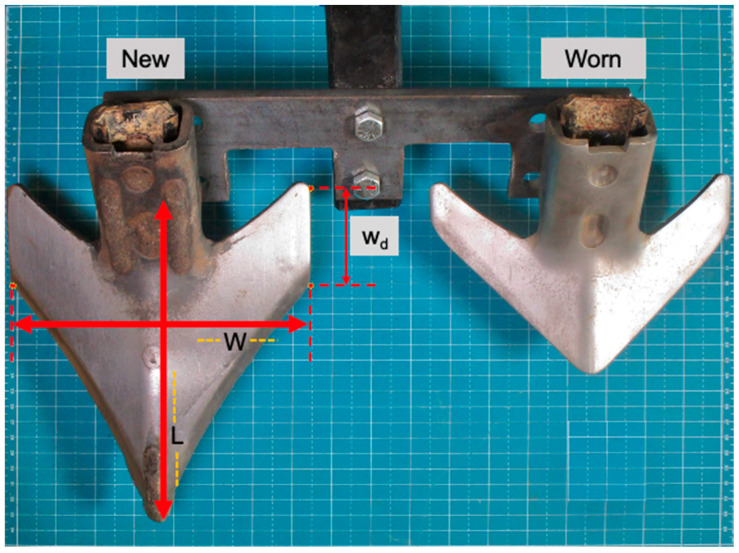
Specifications of the dimensions of the new cultivator sweep (left) and the worn sweep (right). Sweep length (L), sweep front width (W), and wing depth (w_d_) are labeled on the new cultivator sweep. Dotted Lines are marked as boundaries of each dimension, and arrows represent the length covered within those boundaries.

Geometric dimensions (sweep length (L), sweep front width (W), and wing depth (w_d_)), as shown in Figure 3, are measured using a measuring tape (1 mm resolution) on fifty-seven cultivator sweeps from each of the new and worn cultivator sweeps. The new cultivator sweeps are commercially available as Maxxi-Point Field Cultivator Sweep from Case IH equipment manufacturer (Racine, WI, USA). On the Maxxi-Point Field Cultivator Sweep, CADEN Edge coating was applied to the bottom base of the cultivator sweep using tungsten carbide welding to the base of the cultivator sweep, as explained in [17], and are commercially available as Case IH Extenda-Wear products of Agrisolutions (Hamilton, ON, Canada). Case IH provided the fifty-seven worn Case IH Maxxi-point field cultivator sweeps after conducting field wear testing using the Case IH Tiger-Mate 255 Field Cultivator equipped with new Case IH Maxxi-point cultivator sweeps. Tillage tool wear during field cultivation on agricultural soils is caused by low-stress abrasive wear from the soil-to-tool interaction [18].

#### 2.3.1. Specification and Manufacturing Process of Base Cultivator Sweeps

The Case IH Maxxi-Point Field Cultivator Sweep (Racine, WI, USA) was manufactured using hot-rolled coil steel from Manganese Boron alloy, which is similar to commercial grades DIN 30MnB5 and SAE 15B30. Proprietary chemistry and specific steel casting methods, developed by Agrisolutions (Hamilton, ON, Canada), were applied during the manufacturing process, which involves stamping the blank from a coil to a net shape in a flat pattern, followed by heating to full Austenitization temperature for a duration long enough to fully convert the microstructure to gamma-phase Austenite (Agrisolutions). The material is then transferred to a hot-form tooling die and pressed into its fully formed 3D geometry before being directly water-quenched. Finally, it is tempered to achieve a final hardness of 48–52 HRC with all the heat treatment aimed at achieving tempered Martensite microstructure.

#### 2.3.2. Specifications and Manufacturing Process of CADEN Edge Cultivator Sweeps

**Figure 4:**
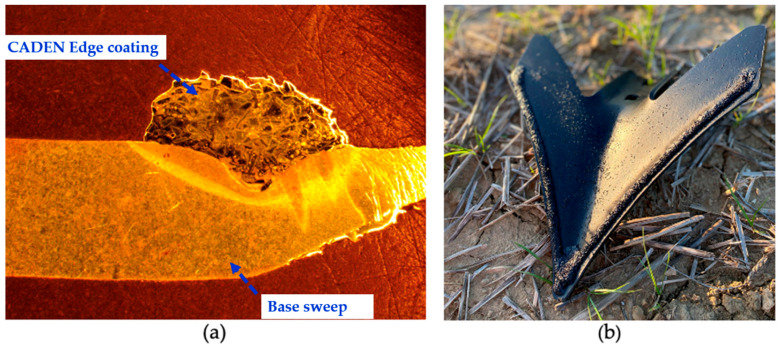
A cross-section of the CADEN Edge coating fused to the base sweep (**a**) and the CADEN Edge-coated polished and etched cultivator sweep (**b**).

The base cultivator sweep (Case IH Maxxi-Point Field Cultivator Sweep) was then coated with a metal matrix composite of alloy steel weld wire and tungsten carbide grit using a gas metal arc welding system to melt the materials onto the wear surface on the sweep (Figure 4). The welding process includes loading the base sweep onto the conveyor, delivering the part to the scanning area using a conveyor, scanning for part location on the conveyor with the Vison system, picking the part by stem or neck with an end-of-arm tooling gripper using an industrial robot, verifying the part location in the gripper, and manipulating the part under a fixed position weld head with an industrial robot employing the GMAW process. Tungsten carbide grit is injected into the molten weld pool with precise velocity and rate to ensure homogenous mixing in the matrix. Finally, the finished parts are dropped into an output chute by the robot.

### 2.4. LiDAR Measurement Setup and Data Collection

The seedbed profiles were measured immediately after tillage operation using SICK LMS511-10100 and SOPAS Engineering Tool acquisition software (Version 2018) (Minneapolis, MN, USA). The loose tilled soil aggregates above the sweep tool depth were carefully removed by hand to expose the seedbed surface after the field cultivator passed. Three static LiDAR data were measured within a designated scanning area (0.77 m wide and 0.83 m long) of the seedbed floor, with a scanning rate of 100 Hz and an angular resolution of 0.5°. The operating manual of SICK LMS511-10100 specifies a statistical error of ±7 mm for distances ranging from 1 to 10 m. No digital filtering (such as fog, echo, or particle filters) from the SOPAS Engineering Tool acquisition software (Version 2018) was applied during the LiDAR scanning. The LiDAR sensor was mounted on the rear of a pickup truck to ensure a consistent distance between the sensor and two black wooden reference plates during data collection. The bottom tip of the SICK LMS511-10100 sensor was approximately 1.5 m from the top undisturbed soil surface. The pickup truck with the LiDAR sensor measurement system was parked in parallel to the tillage direction at each of the sampling position, with the pickup truck center line aligned to the center of the Case IH Tiger-Mate 255 Field Cultivator attached to the Case IH Magnum 380 row crop tractor. For LiDAR soil scanning, three replicates of the tillage treatment plots were randomly selected from the six replicates of the tillage treatment. Each of the three tillage plots was divided into three subplots for taking three static LiDAR scanning data sets within the designated area. A total of fifty-four LiDAR scans (2 tillage treatment × 3 tillage replicates × 3 subplots × 3 scans per designated area) were taken per year. The LiDAR setup and measurement procedures were repeated for each of the three years (2021, 2022, and 2023).

### 2.5. Soil Seedbed LiDAR Scanning and Data Correction

**Figure 5:**
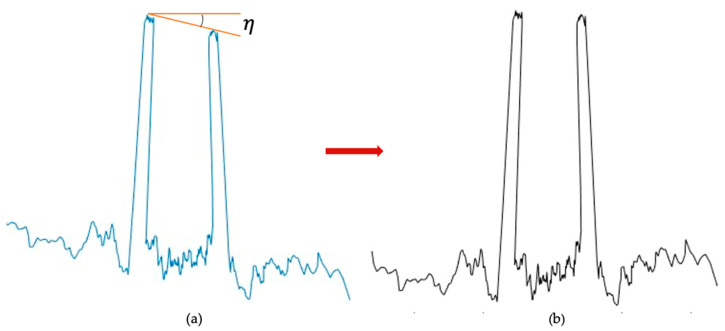
A sample LiDAR-scanned data from the tillage experiment before tilt correction (**a**) and LiDAR-scanned data after correcting for the tilted angle (η) correction (**b**).

The stationary LiDAR scans were analyzed to reconstruct 2D and 3D seedbed surfaces and generate statistical measures of the soil roughness attributes. A MATLAB (MATLAB R2023a, Natick, MA, USA) script was used to produce a soil profile for evaluating soil roughness. Each LiDAR scan consists of 380 points ranging from 5° to 175° (at an interval angle of 0.5°). Data correction was performed in two steps as follows: (1) adjusting for tilting of the LiDAR sensor measurement system due to the wheeling the pickup truck on tilled soil and (2) obtaining the LiDAR data of the seedbed surface height in reference from the two wooden plates close to the tilled soil surface. As the LiDAR sensor and the wooden frame setup are mounted on the back of the pickup truck, there is a potential chance that the pickup truck is slightly tilting due to the truck wheeling on tilled soil surfaces and natural soil relief surfaces (Figure 5a). Correction to the tilting allows to adjust the LiDAR-scanned data to nearly zero angle in the horizontal plane between the two black painted wooden plates (Figure 1). The schematic diagram for correcting LiDAR-scanned data due to tilting (η) is illustrated in Figure 5, showing a sample LiDAR-scanned profile before correction (Figure 5a) and after correction (Figure 5b).

After correcting the LiDAR-scanned data due to tilting, the second step of the data correction process consists of (1) identifying the actual height from the center rotation sensor to soil surface data points and determining the distance from the sensor to reference plates, (2) extracting corrected heights by subtracting the distance from the sensor to reference plates from the actual height, and (3) then extracting the soil height data points between the two reference plates. Details for the data correction steps and their associated mathematical formulas (Equations (1)–(7)) are shown in the following steps:

**Step 1**: Calculate the actual vertical height from the LiDAR sensor to the data points that include reference plates and soil surface corrected by using Equation (1) as follows:

(1) $$h_{i}=lsin\alpha$$

$h_{i}$ is the actual vertical height value from the rotation center of the sensor to the measurement points. l represents the raw distance from the sensor to data points, while α is the angle of each l from the rotation center of the LiDAR sensor.

**Figure 6:**
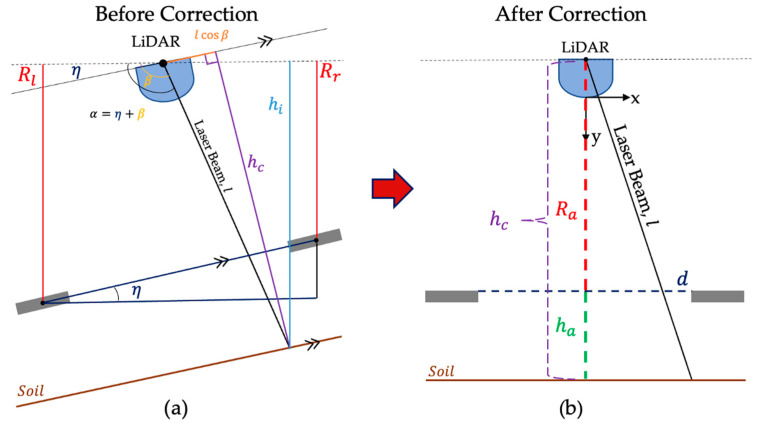
A LiDAR setup diagram before correction of the angle (**a**) and after correction (**b**) using steps 2 to 5. The plates were horizontally leveled by rotating a whole setup in (**a**) by η°. Adjusted heights ($h_{a}$) were used for further analysis of the seed bed profile.

**Step 2**: Correct the tilted angle of the LiDAR based on the height of the two reference plates to adjust the LiDAR-scanned data to a horizontal angle. The vertical height calculated from step 1 requires further correction. As Figure 6b shows, we use two plates on the pickup truck as the reference, which should be on the same height if the LiDAR takes the scanning on a flat surface. The corrected angle (β) is calculated by using Equations (2) and (3) as follows:

(2) $$\begin{array}{c} \eta =\arctan\left(\frac{R_{l}-R_{r}}{d}\right)\; \end{array}$$

(3) β=α−η

$R_{l}$ and $R_{r}$ are the mean actual height of the left and right reference plates, respectively; d is the horizontal distance between the two plates; and η is the estimated tilted angle of LiDAR to the horizontal line. All the collected LiDAR data are corrected by this angle to evaluate soil heights vertically.

**Step 3:** Then the actual vertical height $h_{c}$ is corrected according to the angle β using Equation (4) as follows:

(4) $$\begin{array}{c} h_{c}=l\;sin\beta \end{array}$$

The corrected vertical location is calculated using Equation (5) as follows:

(5) $$\begin{array}{c} x_{a}=xcos\beta \end{array}$$

x is the original vertical location and $x_{a}$ is the location after angle correction.

**Step 4:** Take the average height from the sensor to the reference plates by using Equation (6):

(6) $$\begin{array}{c} R_{a}=\sum_{i=1}^{n}\frac{R_{i}}{n} \end{array}$$

$R_{a}$ is the average height of the two reference plates and $R_{i}$ is the height of point i of the reference plates from the sensor (Figure 6). There are 7 data points for each reference plate (*n* = 7).

**Step 5:** Obtain adjusted height ($h_{a}$) by subtracting the average height of reference plates ($R_{a}$) from the actual vertical height ($h_{c}$) by using Equation (7) as follows:

(7) $$\begin{array}{c} h_{a}=h_{c}-R_{a} \end{array}$$

$h_{a}$ represents the adjusted height, which is the height from reference plates to the soil profile.

After implementing the five steps, the soil height data extracted between the wooden reference frame is used to determine soil roughness attributes. Soil roughness attributes include maximum, minimum, mean, mode, median, standard deviation, coefficient of variation, skewness, roughness, and kurtosis of soil height, which were computed using MATLAB Statistics Toolbox (MATLAB R2023a, Natick, MA, USA). Extracting statistical attributes from processed LiDAR data and analyzing the seedbed soil surface is expected to provide a quantitative assessment of seedbed soil quality for the two-tillage treatment. A smooth seedbed floor (subsurface below the tillage depth) and a uniformly flat, tilled soil seedbed surface are considered optimal seedbed conditions for planting and crop emergence. These conditions establish a favorable soil environment that reduces variability in plant spacing and early plant height, which ultimately contributes to improving crop yield [19]. Examining the statistical distance in the distributions of soil height measurements obtained from the tilled area provides an opportunity to evaluate the uniformity and surface roughness of the seedbed.

### 2.6. Statistical Analysis of Soil Roughness Attributes

**Figure 7:**
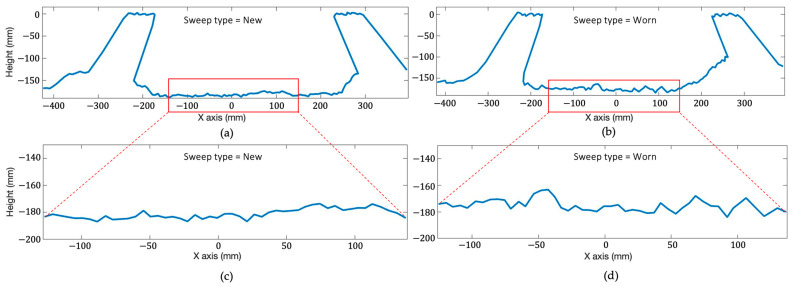
Sample cropped cross-sectional 2D seed bed LiDAR profile from the new coated sweep (**a**) and (**b**) worn sweep tillage treatments. The sample pair of cropped seedbed profiles of (**c**) the new coated sweep and (**d**) worn sweep tillage treatments.

The 2D stationary scans acquired from LiDAR were used to assess the roughness of the seedbed caused by two different tillage tools and to estimate soil roughness within the measurement area [20,21]. The investigation of the cross-sectional area of the seedbed surface is crucial due to the application of two different types of cultivator sweeps to create an even seedbed for optimal crop growth. The 2D seedbed profile was cropped to the most effective zone (covers about 300 mm horizontal distance of the seedbed surface) to achieve better visual representations of the soil roughness of each tillage treatment (Figure 7).

Statistical analysis of soil roughness attributes (maximum(max), minimum(min) mean, median, mode, standard deviation, coefficient of variation, kurtosis, skewness and roughness coefficient) of corrected height data from each scanning area were collected and compared to investigate the impacts of two tillage tools (new and worn cultivator sweeps). Roughness coefficient (maximum range divided by the mean) was calculated after Tekeste et al. (2024) [22]. For statistical comparison of the soil roughness attributes by the tillage treatment, we used Analysis of Variance (ANOVA), Kolmogorov–Smirnov (KS) Test, and Earth Mover’s Distance method.

#### 2.6.1. Analysis of Variance (ANOVA)

An Analysis of Variance (ANOVA) test was used to assess how well the soil roughness attributes represented the effects of the two different tillage tool types. In our experiment, we describe the ANOVA model as follows:

(8) $$\begin{array}{c} y_{ijkl}=\mu +\alpha_{i}+\beta_{j}+{\alpha \beta }_{ij}+\varepsilon_{ijkl}, \end{array}$$

where μ is the global mean of the soil roughness parameter, $\alpha_{i}$ is the fixed treatment effect of using new or worn sweeps, $\beta_{j}$ is the random block effect of different subplot, ${\alpha \beta }_{ij}$ represents the interaction effect of the treatment and the block, and $\varepsilon_{ijkl}$ is the normally distributed random error.

Then, we calculate the F-statistic for the interested effect by the mean squares of each effect to the mean square error and compare the calculated F-statistics to critical values from the F-distribution. If the F-statistic for a particular effect is larger than the critical value, it indicates that the effect is statistically significant. A *p*-value of 0.05 is used to determine the statistical significance.

#### 2.6.2. Two-Sample Kolmogorov–Smirnov (KS) Test

The Kolmogorov–Smirnov (KS) test is the most well-known test for normality, and is included in many statistical software packages. As a nonparametric test of the equality of discontinuous, the KS test qualifies a distance between the cumulative distribution function of the reference distribution and provides a *p*-value. The null hypothesis of this test is that the sample is drawn from the same distribution. The two-sample KS test is one of the most useful methods for comparing two samples, as it has the capability to detect differences in both the location and shape of the empirical cumulative distribution functions of two samples. The KS distance is always between 0 and 1 [23].

The KS test for comparing two empirical distributions P with samples $P_{1},\ldots ,P_{n}$ and Q with samples $Q_{1},\ldots ,Q_{m}$, is defined as follows:

(9) $$\begin{array}{c} D_{m,n}=\underset{i,j}{\max}\left|P_{i}-Q_{j}\right|,\;1\leq i\leq n,1\leq j\leq m, \end{array}$$

where $D_{m,n}$ follows the Kolmogorov distribution distance value.

#### 2.6.3. Earth Mover’s Distance Method

The Earth Mover’s Distance (EMD) method, also known as the Wasserstein distance, is a method to evaluate dissimilarity between two multidimensional distributions. Using the EMD method, we compared the distributions of soil height from each tillage treatment (new and worn sweep). In the context of comparing two distributions, the EMD measures the amount of work distance required to transform one distribution to another and is often a useful method for measuring their dissimilarity quantitatively. Unlike the Kolmogorov–Smirnov test, the EMD is unbounded.

Formally, if we have two empirical distributions P with samples $P_{1},\ldots ,P_{n}$ and Q with samples $Q_{1},\ldots ,Q_{n}$, then the EMD is defined as follows:

(10) $$\begin{array}{c} EMD\left(P,Q\right)={\left(\frac{1}{n}{\sum_{i}\left|\left|X_{\left(i\right)}-Y_{\left(i\right)}\right|\right|}^{p}\right)}^{\frac{1}{p}}, \end{array}$$

where $X_{\left(i\right)}\;and\;Y_{\left(i\right)}$ are the order statistics for P and Q, respectively. We use p=2 for the two tillage treatments.

## 3. Results and Discussion

### 3.1. Initial Soil Conditions

The initial soil moisture data collected from the topsoil sample for each year at each LiDAR measurement area are shown in Table 1. The smaller magnitude of the standard deviation of initial soil moisture (0.91% on a dry basis) suggests that there was a minimum effect on tillage due to soil moisture content variation within the plots and among the three years.

In May, when the tillage was conducted, the cumulative precipitation was 77 mm (2021), 88 mm (2022), and 85 mm (2023) with a standard deviation of 5 mm for three years. The cumulative monthly precipitation amounts were typical spring rainfall in the study area.

### 3.2. Sweeps Wear Characteristics

The mean mass loss of the worn sweeps was 40% relative to the new sweeps (Table 2). The mean dimensions losses of the sweep without hardening as compared to the CADEN edge hardened sweeps (new sweeps) were 32% (sweep length), 58% (sweep wing depth), and 33% (sweep front width). The highest wear loss of 58% was shown in the cutting wing depth of the sweep, resulting in a worn sweep magnitude of 36 mm.

### 3.3. 2D and 3D Generated Soil Seedbed Profiles

**Figure 8:**
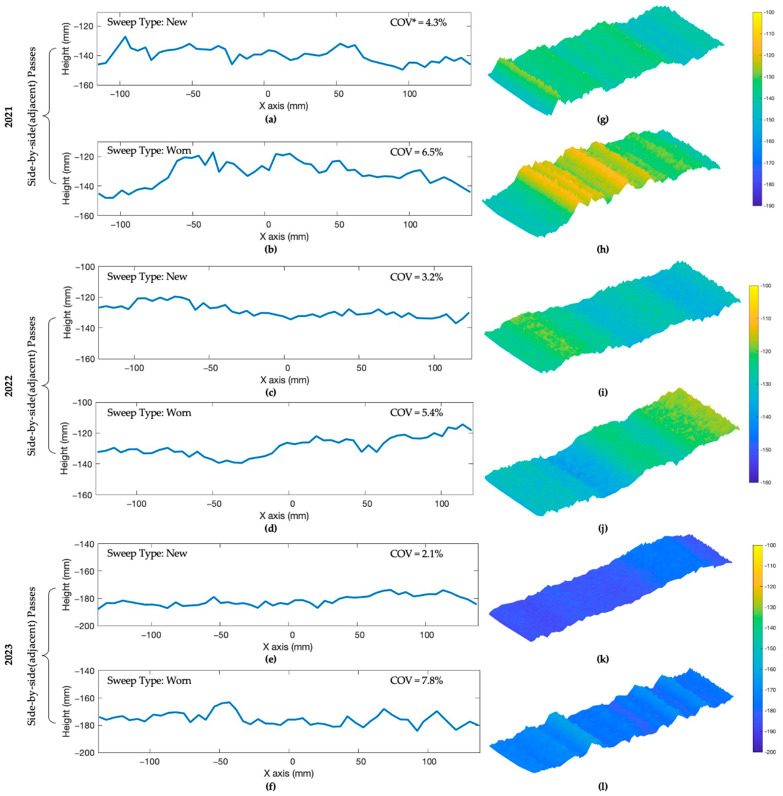
A sample pair of 2D scans of new coated sweep (**a**,**c**,**e**) and worn sweep (**b**,**d**,**f**) tillage treatment from side-by-side passes and 3D representation scans of new (**g**,**i**,**k**) and worn (**h**,**j**,**l**) tillage treatment with COV* (coefficient of variation) above from each experimental year (from 2021 to 2023).

Each 2D cropped profile was converted to 3D visuals based on the number of slices measured on each measurement area. The 3D-generated profile of LiDAR scans provided a detailed representation of the soil profile, allowing for a more intuitive visual analysis of its structure and soil variation. This combination overcomes limitations in traditional measurement, which often rely on subjective observations and manual measurements. A repeated sample pair of collected 2D data from 2021, 2022, and 2023 were selected for visual comparison (Figure 8).

The 2D and 3D scans were generated based on static LiDAR scans of the bottom soil surface after tillage and removing the tilled loose soil. The subfigures (Figure 8a–f) represent pairs of 2D profiles from new and worn cultivator sweeps treatment obtained from adjacent passes. The subfigures (Figure 8g–l) show the corresponding 3D profiles of the same sweeps. Upon inspecting the visual representation, it showed the new sweeps produce a more even seedbed profile compared to the worn sweeps when comparing two tillage treatments. The coefficient of variation (COV) was calculated by dividing the population standard deviation by the population mean on sample 2D profiles. The visual figure of the seedbed profile created by new coated sweeps showed lower COV than worn sweeps. This indicates that LiDAR showed applicability to perform on-the-go measurements of the soil surface in field conditions [20] and that tillage tool wear affects the formation of the smooth seedbed (Figure 8).

### 3.4. Seedbed Roughness Measurement

#### 3.4.1. Distribution Analysis in Soil Roughness Using Earth Mover’s Distance (EMD) and Kolmogorov–Smirnov (KS) Tests

The Earth Mover’s Distance (EMD) test, a statistical measure that quantifies the minimum distance needed to transform one distribution into another, shows the comparison of the overall shape and distribution of soil height distribution factors between the two tillage treatments. The evaluation of EMD results explains the dissimilarity distance value between the two tillage treatments and within treatments since there is no threshold value to statistically differentiate features (Table 3).

The mean minimum distance of work between treatments resulted from the EMD test showed an average dissimilarity of 4% greater than the distribution within treatments over three years (Table 3). The data suggests that the EMD test is capable of detecting differences in the distribution shape by measuring the minimum work distance from different shapes of the distribution of mean soil height data from the LiDAR data set between the two tillage treatments. By comparing the minimum work distance of distributions, EMD could be useful to adapt as a method to classify two tillage treatments. However, it is hard to address the statistical differences between two tillage treatments because there is no threshold value.

**Figure 9:**
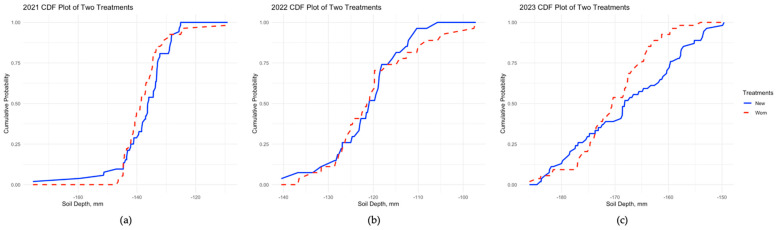
CDF (cumulative distribution function) plot of mean soil heights (or depths) from new (blue line) and worn (red line) cultivator sweeps from 2021 (**a**), 2022 (**b**), and 2023 (**c**).

Utilizing the two-sample Kolmogorov–Smirnov (KS) test showed the differences in soil roughness distribution between the two tillage treatments. In Figure 9, cumulative distribution function (CDF) plots of the KS test from the three years of data illustrate the differences in mean soil height distribution from the two tillage treatments. The results of the KS test showed that there was a significant difference (*p* < 0.05) in the mean soil height and skewness distribution of soil roughness between the two tillage treatments in 2021 and 2023, but not from the data collected in 2022 (Figure 9). In one of the three years of study, the other statistical variables, such as median, mode standard deviation of soil height, and kurtosis of soil height distribution, were statistically different (*p* < 0.05) between the two tillage treatments (Table 4).

#### 3.4.2. Soil Roughness Attributes ANOVA Analysis

Mean value comparison between the two tillage treatments on soil roughness attributes (maximum, minimum, mean, median, mode, standard deviation (Std), COV skewness, kurtosis, and roughness coefficient) is shown in Table 5.

The maximum, minimum, mean, and median height (depth from reference plates) of the soil profiles, observed in 2021 and 2022 (not in 2023), revealed that new cultivator sweeps produced deeper seedbed profiles compared to worn cultivator sweeps. By comparing the standard deviation of height [10], it was observed that the worn cultivator sweeps resulted in higher values than new sweeps by 17.8%, 2.1%, and 7.9% for three years of study (Table 5). The standard deviation and coefficient of variation of soil height showed consistently higher values in worn cultivator sweeps, indicating more soil disturbance in the seedbed profile formed by worn cultivator sweeps. The kurtosis value was higher for the new cultivator sweeps compared to worn sweeps, indicating a more peaked and concentrated distribution of mean soil height values (Table 5). This indicates that worn sweep tillage treatment led to a broader distribution of mean soil heights, resulting in a wider range of soil height distribution on the seedbed profile. However, more experiments under different soil types to investigate their effects on seedbed formation are needed to fully understand the effects.

The ANOVA results shown in Table 6 also show ANOVA effects, including tillage treatment ($\alpha_{i}$) and subplot as fixed factors, and cross effect ($\alpha \beta_{ij}$) that were employed to analyze the soil roughness factors obtained from LiDAR data (Table 7).

Based on the results of the ANOVA, significant differences were observed in the cross effect of tillage treatment and subplot for three years with respect to COV and Std among soil roughness factors. This suggests that comparisons of soil tilth should take into consideration both tillage treatment and different soil types if they are based on Std and COV. By comparing the ANOVA outcomes for kurtosis of the distribution of mean height from each tillage treatment, it became evident that significant differences were observed between two tillage treatments only with regards to treatment effects in two out of three years. This suggests that kurtosis could serve as a valuable measure for assessing soil tilth quality, even across different soil types. F-statistics estimates and *p*-values from the ANOVA test are listed in Table 7.

This study demonstrates that tillage wear resulted in losses in mass and dimensional parameters of seedbed cultivator sweeps. The relative magnitude of mass loss of cultivator sweeps used for the field study (mass wear loss of 40%) appears to be similar to the mass wear loss (30%) reported in [15] from controlled circular wear testing. Cultivator sweeps are worn most on the sweep wing depth (Wd loss of 58%) as compared to the other measured dimensions (sweep length and sweep front wing width). The worn cultivator sweep had a 61 mm smaller sweep front wing compared to the sweep frontal wing of the new CADEN edge hardened cultivator sweep. Such sweep frontal wing wear loss (61 mm) was 2.4 times the recommended overlap of the adjacent sweeps (25.4 mm) according to the manual Case IH Tiger-Mate 255 Field Cultivator. The wear losses on the sweep wing dimensions (Wd loss of 58% and W loss of 33%) might have contributed to the uneven seedbed surface across the measured, as evidenced by the LiDAR data analytics after field cultivation using the worn sweeps. These sweep wing dimensional losses might have contributed to the statistically significant effects of the tillage treatments on soil roughness spread descriptors such as standard deviation, coefficient of variance, kurtosis, and roughness index, parameters that seem to describe the uneven seedbed surface across the width of the LiDAR-scanned area after a worn cultivator sweep pass. The tillage wear-induced loss of sweep length appears to have caused the statistically significant differences in the maximum soil roughness estimates. Using the CADEN Edge hardened cultivator sweeps for seedbed tillage might reduce the losses in wear-induced uneven seedbed surfaces and maintain the desired overlap (25.4 mm) between the adjacent cultivator sweeps of the field cultivator equipment. The three-year seedbed tillage study demonstrated applying hard facing (CADEN Edge using tungsten carbide grit) on cultivator sweeps created relatively uniform seedbed quality for better planting outcomes and crop yield. Creating favorable seedbeds with hard-faced cultivator sweeps provides benefits to growers besides the enhanced longevity of the cultivator sweeps for field tillage operations and improved field efficiency reported from a previous study [15]. According to [22] up to half a million (567,996) unhardened cultivator sweeps are expected to be replaced after experiencing 30% mass wear loss, assuming a USDA-estimated 83 million hectares of crop field received tillage before planting every year. Besides achieving better seedbed quality, CADEN Edge hardened sweeps also provide economic benefits by extending the replacement cycle of cultivator sweeps. Implementing soil roughness analytics from the LiDAR-generated parametric and nonparametric statistical analysis demonstrates that digited seedbed tillage assessment is better than the time-consuming and error-prone manual pinboard and chain methods. The statistical methods (ANOVA, KS, and EMD) deployed for quantifying the seedbed surface quality differences due to the cultivator sweep life cycle could be integrated to develop a feedback algorithm to the AFS Soil command sensing and control of the field cultivator equipment (Case IH Tiger-Mate 255 Field Cultivator) used in our study or integrated as real-time seedbed quality assessment into advanced automation of smart tillage processes [24].

## 4. Conclusions

The research evaluated seedbed quality using LiDAR technology and investigated the impact of tillage tool wear on seedbed quality by a designed field experiment. We visualized LiDAR data-generated 2D and 3D representations for evaluating the quality of seedbeds using new and worn cultivator sweeps. Three different statistical methods (ANOVA, KS, and EMD) were compared on seedbed soil roughness attributes created by new and used cultivator sweeps.

The following conclusions were drawn from soil tilth quality comparisons from new and worn cultivator sweeps using LiDAR sensor technology:

1. ANOVA results between new and worn sweep tillage treatment data showed significant differences (*p* < 0.05) on soil roughness variables (standard deviation coefficient of variation and kurtosis) with interaction effects of subplot soil type and sweep treatment from 2021 to 2023 data and main sweep treatment effect from 2021 data. Kurtosis of the mean height from LiDAR data could be used as a potential factor to compare soil quality.
2. The KS test, a comparison of soil tilth distribution, especially in mean soil height and skewness data, showed statistically significant differences (*p* < 0.05) between the two tillage treatments in all subplot soil in 2021 and 2023 data.
3. According to the EMD measure of dissimilarity, several pairwise distributions between the new and worn sweeps showed an average of 4% difference in three years, demonstrating the capability to classify two tillage treatments.
4. This study concludes that tillage tool wear substantively affects seedbed quality, as evidenced by varying soil roughness factors. Our study supports the adoption of LiDAR technology for seedbed management, highlighting its applicability to evaluate seedbed quality. This research provides valuable insights into how tillage tool wear affects seedbed quality and supports crop growers in making better decisions about tillage management. Further research and more experiments are needed to develop the proposed LiDAR sensing techniques for comparing seedbed tilth quality of the LiDAR data in different soil types and long-term effects on crop yield and farm economics.

## Figures and Tables

**Table 1:** The mean, standard deviation, and coefficient of variation of soil moisture content from the soil sample from each measurement area. Sample size for the soil sampling was 18.

Soil Moisture Content (Dry Basis (d.b.), %) *			
	Year		
Parameters	2021	2022	2023
Mean	14.66%	15.64%	16.48%
Std	1.94%	1.72%	0.96%
COV	13.24%	10.99%	5.81%

* Topsoil samples (approximately 0 to 102 mm) were collected after tillage operation and at the same area where the LiDAR measurements were taken. The samples were oven-dried at 107° for 72 h to determine soil moisture content on a dry basis. The measured mean moisture content at lower plastic limit (PL) was 24.49% (d.b.), std = 1.50% (d.b.), COV = 6.12%, and N = 9, and the mean moisture content at liquid limit (LL) was 31.52% (d.b.), std = 1.32% (d.b.), COV = 4.18%, and N = 3.

**Table 2:** Mass and geometric dimensions of the new and worn cultivator sweeps.

Parameters for Sweep Type ^[a]^	Sweep Length (L) (mm)	Sweep Wing Depth (W_d_) (mm)	Sweep Front Width (W) (mm)	Sweep Mass (g)
New				
Mean	243	63	184	1579
Std	2	2	3	35
CoV	1%	3%	2%	2%
Worn				
Mean	166	27	123	946
Std	20	15	23	164
CoV	12%	56%	18%	17%

^[a]^ The mean, std, and COV of each dimension were estimated from the cultivator sweep specimens (*n* = 56). The sweep dimension features of sweep length (L), wing depth (Wd), and front width (W) are shown in Figure 3.

**Table 3:** The mean minimum distance of work to move from one distribution to another. Within treatments, it indicates new sweep versus new sweep or worn sweep versus worn sweep. Between treatments, it compares the distance between two tillage treatments.

Year	Within Treatments	Between Treatments	% Difference
2021	8.76	9.09	3%
2022	9.78	9.99	1%
2023	10.59	11.22	4%
Three-year mean	9.71	10.10	3%

**Table 4:** A *p*-value table of the KS test for statistical variables between two tillage treatments.

Year	Maximum	Minimum	Mean	Median	Mode	Std	COV	Skewness	Kurtosis	Roughness Coefficient
2021	0.49	0.20	0.04 *	0.04 *	0.001 *	0.10	0.07	<0.01 *	<0.01 *	0.13
2022	0.75	1.00	0.75	0.93	0.93	0.18	0.52	0.18	0.10	0.32
2023	0.09	0.05	0.03 *	0.08	0.05	0.03 *	0.52	0.01 *	0.21	0.08

* Indication represents the statistically significant difference by p-value<0.05.

**Table 5:** Statistical variables of soil height from seedbed profile data including maximum, minimum, mean, median, mode, Std, COV of height, skewness, kurtosis, and roughness coefficient from static LiDAR scan data.

Year, Sweep Type	Maximum of Height (mm)	Minimum of Height (mm)	Mean Height (mm)	Median Height (mm)	Mode of Height (mm)	Std of Height (mm)	COV of Height	Skewness	Kurtosis	Roughness Coefficient
2021										
New	−125.93	−152.83	−140.23	−140.89	−140.33	5.92	4.23%	0.08	2.67	−0.19
Worn	−120.09	−149.88	−136.02	−137.62	−136.42	7.08	5.33%	0.14	2.35	−0.22
2022										
New	−106.90	−134.21	−121.44	−122.41	−121.81	6.22	5.11%	0.17	2.70	−0.22
Worn	−106.26	−133.50	−120.21	−120.49	−120.30	6.35	5.34%	0.05	2.50	−0.23
2023										
New	−147.99	−184.21	−172.38	−173.77	−175.43	7.70	4.49%	1.13	4.48	−0.21
Worn	−153.78	−189.03	−174.27	−175.09	−175.55	8.33	4.80%	0.35	3.23	−0.20

**Table 6:** A *p*-value table of ANOVA test results by tillage treatment ($\alpha_{i}$) and cross effects of tillage treatment and subplot ($\alpha \beta_{ij}$).

Year, Fixed Effects	Maximum of Height	Minimum of Height	Mean Height	Median Height	Mode of Height	Std of Height	COV of Height	Skewness	Kurtosis	Roughness Coefficient
2021										
$\alpha_{i}$	0.042 *	0.180	0.092	0.128	0.243	0.025 *	0.018 *	0.403	0.001 *	0.078
$\alpha \beta_{ij}$	0.032 *	0.315	0.108	0.166	0.342	0.021 *	0.016 *	0.031 *	0.170	0.045 *
2022										
$\alpha_{i}$	0.756	0.738	0.522	0.436	0.338	0.715	0.435	0.260	0.057	0.687
$\alpha \beta_{ij}$	0.005 *	0.352	0.128	0.162	0.260	0.013 *	0.019 *	0.238	0.257	0.001 *
2023										
$\alpha_{i}$	0.090	0.012 *	0.275	0.437	0.948	0.282	0.370	0.000 *	0.012 *	0.625
$\alpha \beta_{ij}$	0.059	0.026 *	0.035 *	0.038 *	0.073	0.013 *	0.014 *	0.599	0.779	0.160

* Indication represents significance *p*-value <0.05.

**Table 7:** Summary of ANOVA test estimates of LiDAR data by tillage treatment ($\alpha_{i}$) effects and cross effects of tillage treatment and subplot ($\alpha \beta_{ij}$) over three years with degree of freedom (df), sum of squares, *F*-value, and *p*-value.

Variables	Effects	df	Sum Square	*F*-Value	*p*-Value
2021					
Maximum	$\alpha_{i}$	1	460.64	4.32	0.042 *
	$\alpha \beta_{ij}$	2	781.52	3.67	0.032 *
Minimum	$\alpha_{i}$	1	118.14	1.84	0.180
	$\alpha \beta_{ij}$	2	151.08	1.18	0.315
Mean	$\alpha_{i}$	1	239.60	2.93	0.092
	$\alpha \beta_{ij}$	2	380.26	2.33	0.108
Median	$\alpha_{i}$	1	206.01	2.93	0.092
	$\alpha \beta_{ij}$	2	320.71	1.86	0.166
Mode	$\alpha_{i}$	1	144.22	1.39	0.243
	$\alpha \beta_{ij}$	2	226.09	1.09	0.342
Std	$\alpha_{i}$	1	18.13	5.33	0.025 *
	$\alpha \beta_{ij}$	2	28.29	4.15	0.021
COV	$\alpha_{i}$	1	<0.01	5.90	0.018 *
	$\alpha \beta_{ij}$	2	<0.01	4.46	0.016 *
Skewness	$\alpha_{i}$	1	0.06	0.70	0.403
	$\alpha \beta_{ij}$	2	0.63	3.72	0.031 *
Kurtosis	$\alpha_{i}$	1	1.31	10.96	0.001 *
	$\alpha \beta_{ij}$	2	0.43	1.83	0.170
Roughness coefficient	$\alpha_{i}$	1	0.01	3.23	0.07
	$\alpha \beta_{ij}$	2	0.02	3.29	0.04 *
**Variables**	**Effects**	**df**	**Sum Square**	***F*** **-Value**	***p*** **-Value**
**2022**					
Maximum	$\alpha_{i}$	1	5.52	0.09	0.756
	$\alpha \beta_{ij}$	2	649.50	5.73	0.005 *
Minimum	$\alpha_{i}$	1	6.77	0.11	0.738
	$\alpha \beta_{ij}$	2	128.36	1.06	0.352
Mean	$\alpha_{i}$	1	20.55	0.41	0.522
	$\alpha \beta_{ij}$	2	212.21	2.14	0.128
Median	$\alpha_{i}$	1	30.75	0.61	0.435
	$\alpha \beta_{ij}$	2	187.51	1.88	0.162
Mode	$\alpha_{i}$	1	49.75	0.93	0.338
	$\alpha \beta_{ij}$	2	147.54	1.38	0.260
Std	$\alpha_{i}$	1	0.22	0.13	0.715
	$\alpha \beta_{ij}$	2	16.23	4.74	0.013 *
COV	$\alpha_{i}$	1	<0.01	0.61	0.435
	$\alpha \beta_{ij}$	2	<0.01	7.13	0.001 *
Skewness	$\alpha_{i}$	1	0.18	1.29	0.260
	$\alpha \beta_{ij}$	2	0.43	1.47	0.238
Kurtosis	$\alpha_{i}$	1	0.52	3.79	0.057
	$\alpha \beta_{ij}$	2	0.38	1.39	0.257
Roughness coefficient	$\alpha_{i}$	1	0.00	0.16	0.68
	$\alpha \beta_{ij}$	2	0.02	7.47	0.001
**Variables**	**Effects**	**df**	**Sum Square**	***F*** **-value**	***p*** **-value**
**2023**					
Maximum	$\alpha_{i}$	1	452.45	2.98	0.090
	$\alpha \beta_{ij}$	2	907.45	2.99	0.059
Minimum	$\alpha_{i}$	1	313.07	6.81	0.012 *
	$\alpha \beta_{ij}$	2	359.62	3.91	0.026 *
Mean	$\alpha_{i}$	1	48.08	1.21	0.275
	$\alpha \beta_{ij}$	2	283.63	3.58	0.035 *
Median	$\alpha_{i}$	1	23.62	0.61	0.437
	$\alpha \beta_{ij}$	2	270.06	3.49	0.038 *
Mode	$\alpha_{i}$	1	0.18	<0.01	0.948
	$\alpha \beta_{ij}$	2	239.08	2.75	0.073
Std	$\alpha_{i}$	1	5.30	1.18	0.282
	$\alpha \beta_{ij}$	2	42.03	4.68	0.013 *
COV	$\alpha_{i}$	1	<0.01	0.81	0.370
	$\alpha \beta_{ij}$	2	<0.01	4.60	0.014 *
Skewness	$\alpha_{i}$	1	8.21	16.42	<0.01 *
	$\alpha \beta_{ij}$	2	0.51	0.51	0.599
Kurtosis	$\alpha_{i}$	1	21.31	6.76	0.012 *
	$\alpha \beta_{ij}$	2	1.57	0.25	0.779
Roughness coefficient	$\alpha_{i}$	1	<0.01	0.24	0.625
	$\alpha \beta_{ij}$	2	0.01	1.9	0.160

* Indication represents significance *p*-value <0.05.

## Data Availability

Data are contained within the article.

## References

[1] Karayel D., Özmerzi A. (2002). Effect of tillage methods on sowing uniformity of maize. Candian Biosyst. Eng..

[2] Sandri R., Anken T., Hilfiker T., Sartori L., Bollhalder H. (1998). Comparison of methods for determining cloddiness in seedbed preparation. Soil Tillage Res..

[3] Colvin T.S., Erbach D.C., Buchele W.F., Cruse R.M. (1984). Tillage index based on created soil conditions. Trans. ASAE.

[4] Kuipers H. (1957). A reliefmeter for soil cultivation studies. Neth. J. Agric. Sci..

[5] Saleh A. (1993). Soil roughness measurement: Chain method. J. Soil Water Conserv..

[6] Wolock D.M., Price C.V. (1994). Effects of digital elevation model map scale and data resolution on a topography-based watershed model. Water Resour. Res..

[7] Ball B.C., Guimarães R.M.L., Cloy J.M., Hargreaves P.R., Shepherd T.G., McKenzie B.M. (2017). Visual soil evaluation: A summary of some applications and potential developments for agriculture. Soil Tillage Res..

[8] Milenkovic M., Pfeifer N., Glira P. (2015). Applying terrestrial laser scanning for soil surface roughness assessment. Remote Sens..

[9] Thomsen L.M., Baartman J.E.M., Barneveld R.J., Starkloff T., Stolte J. (2015). Soil surface roughness: Comparing old and new measuring methods and application in a soil erosion model. Soil.

[10] Foldager F.F., Pedersen J.M., Skov E.H., Evgrafova A., Green O. (2019). LiDAR-Based 3D Scans of Soil Surfaces and Furrows in Two Soil Types. Sensors.

[11] Fanigliulo R., Antonucci F., Figorilli S., Pochi D., Pallottino F., Fornaciari L., Grilli R., Costa C. (2020). Light drone-based application to assess soil tillage quality parameters. Sensors.

[12] Oz I., Arav R., Filin S., Assouline S., Furman A. (2017). High-resolution measurement of topographic changes in agricultural soils. Vadose Zone J..

[13] Znova L., Melander B., Lisowski A., Klonowski J., Chlebowski J., Edwards G.T., Nielsen S.K., Green O. (2018). A new hoe share design for weed control: Measurements of soil movement and draught forces during operation. Acta Agric. Scand. Sect. B—Soil Plant Sci..

[14] Natsis A., Papadakis G., Pitsilis J. (1999). The influence of soil type, soil water and share sharpness of a mouldboard plough on energy consumption, rate of work and tillage quality. J. Agric. Eng. Res..

[15] Tekeste M.Z., Balvanz L.R., Al-Aani F., Boesenberg A., Hatfield J.L. (2022). Hardened Edges Effects on Wear Characteristics of Cultivator Sweeps Using Circular Soil Bin Test. J. Tribol..

[16] Mann P.S., Brar N.K. (2015). Tribological aspects of agricultural equipment: A review. Int. Res. J. Eng. Technol..

[17] Tekeste M.Z., Balvanz L.R., Hatfield J.L., Ghorbani S. (2019). Discrete element modeling of cultivator sweep-to-soil interaction: Worn and hardened edges effects on soil-tool forces and soil flow. J. Terramechanics.

[18] Swanson P.A., Ruff A.W., Bayer R.G. (1993). Comparison of Laboratory Abrasion Tests and Field Tests of Materials Used in Tillage. Tribology: Wear Test Selection for Design and Application.

[19] Nielsen R.L. (2004). Effect of Plant Spacing Variability on Corn Grain Yield.

[20] Jensen T., Green O., Munkholm L.J., Karstoft H. (2016). Fourier and granulometry methods on 3D images of soil surfaces for evaluating soil aggregate size distribution. Appl. Eng. Agric..

[21] Jensen T., Munkholm L.J., Green O., Karstoft H. A mobile surface scanner for soil studies. Proceedings of the Second International Conference on Robotics, Associated High-Technologies and Equipment for Agriculture and Forestry-RHEA 2014.

[22] Tekeste M.Z., Guo J., Habtezgi D., He J.-H., Waz M. (2024). Development of a Method for Soil Tilth Quality Evaluation from Crumbling Roller Baskets Using Deep Machine Learning Models. Sensors.

[23] Berger V.W., Zhou Y. (2014). Kolmogorov–Smirnov Test: Overview. Wiley Statsref: Statistics Reference Online.

[24] Boysen J., Zender L., Stein A. (2023). Modeling the soil-machine response of secondary tillage: A deep learning approach. Smart Agric. Technol..

